# Decision-Making Impairments in Patients with Parkinson’s Disease

**DOI:** 10.1155/2004/578354

**Published:** 2005-01-27

**Authors:** Matthias Brand, Kirsten Labudda, Elke Kalbe, Rüdiger Hilker, David Emmans, Gerd Fuchs, Josef Kessler, Hans J. Markowitsch

**Affiliations:** ^1^Department of Physiological PsychologyUniversity of BielefeldBielefeldGermany; ^2^Max-Planck-Institute for Neurological ResearchCologneGermany; ^3^Department of NeurologyUniversity HospitalCologneGermany; ^4^Parkinson Clinic WolfachGermany

**Keywords:** gambling, executive functions, feedback processing, dorsolateral prefrontal cortex, orbitofrontal cortex

## Abstract

A high percentage of Parkinson’s disease (PD) patients show cognitive impairments in addition to the cardinal motor symptoms. These deficits primarily concern executive functions most probably linked to dysfunctions in prefrontal regions due to decreased dopaminergic transmission in fronto-striatal loops. To investigate possible associations between decision-making and executive functions in PD, we examined 20 non-demented PD patients and 20 healthy control subjects with a neuropsychological test battery and the Game of Dice Task. In this computerised decision-making task, the rules for gains and losses and the winning probabilities are obvious and stable. Thus, strategic components besides feedback processing might influence decision-making in this task. We found that PD patients were impaired in the Game of Dice task performance and that the frequency of disadvantageous choices correlated with both executive functions and feedback processing. We suggest that decision-making deficits of PD patients in explicit gambling situations might be associated with dysfunctions in two different fronto-striatal loops: the limbic-orbitofrontal-striatal loop, involved in feedback processing, and the dorsolateral prefrontal-striatal loop, involved in executive functions.

## 1. Introduction

Parkinson's disease (PD) is primarily characterised by the cardinal motor symptoms of rigor, tremor, and bradykinesia. Beyond these motor impairments, depression and other psychiatric symptoms [37] as well as cognitive dysfunctions often occur in the course of PD (e.g. [26]). In addition to a general decline of information processing speed [9] the most prominent cognitive deficits of PD patients comprise disturbances of executive functions such as cognitive flexibility, strategy learning and application, working memory and different forms of higher order attention and interference susceptibility [4,17,42,46,50]. Furthermore, anterograde memory impairments [53] and a decline of social skills – shown for instance in a 'theory of mind'-paradigm [44] – have also been reported. Functional disturbances of fronto-striatal loops resulting from a deficient dopamine transmission due to cell loss within the substantia nigra is considered the primary neural correlate for cognitive deficits in PD [13,27,34,43]. Given that one of the main neurochemical correlates of pathological gambling is also a dopaminergic alteration [41], some authors assume that patients with PD show a higher rate of pathological gambling symptoms potentially dependent on disease duration and dopaminergic medication [18,23,38,45]. However, the question remains whether PD patients without clinically relevant symptoms of pathological gambling already show decision-making impairments with a tendency towards

ISSN 0953-4180/04/$17.00 © 2004 – IOS Press and the authors. All rights reserved risky decisions as patients with pathological gambling do [15].

Thiel et al. [51] examined a small group of five PD patients with a computerised version of the Iowa Gambling Task [5], which is probably the most frequently used task to assess decision-making. This task offers implicit rules for gains and losses associated with different card decks and therefore decisions under ambiguity. Thiel et al. conducted $^{18}$F-2-fluoro-2-deoxy-D-glucose positron emission tomography ($^{18}$FDG-PET) while participants' performance of the Iowa Gambling Task. They revealed that the patients showed less activation of the orbitofrontal cortex and a thalamic deactivation compared to healthy control subjects during Iowa Gambling Task performance. The authors suggested that the so-called limbic loop [1,2] – linking the mesial orbitofrontal cortex and the anterior cingulate gyrus to the ventral striatum (nucleus accumbens which is also connected with the substantia nigra and the ventral pallidum) – seems to be dysfunctional. In contrast, the 'cognitive loop' [1,2] – mainly comprising the dorsolateral prefrontal cortex and lateral orbitofrontal cortex with the caudate nucleus which is directly and indirectly linked to the substantia nigra – was suggested to be less affected in at least early or moderate stages of the disease. In two consecutive investigations Czernecki et al. [16] studied levodopa treated PD patients with the Iowa Gambling Task and found that the patients performed the task in a similar manner compared to the control subjects during the first assessment (see also [48]), but did not profit from repetition to the degree controls did. The authors argued that in PD a decrease of motivation or an insensitivity for reinforcement influences attentional processes required to learn the alternative-reinforcement and alternative-punishment associations.

However, the question remains whether PD patients are impaired in a gambling task with explicit rules involving strategy application (i.e. executive functions) as well as feedback processing. In addition, it is not yet clarified whether possible decision-making deficits in such a risk-taking decision-making task are associated with executive dysfunctions and/ or disturbances of feedback processing (in terms of processing gains and losses) in PD patients. In the present study, we examined PD patients with a risk-taking decision-making task with explicit rules and hypothesised that PD patients are deficient due to both, executive dysfunctions and abnormalities in the use of emotional feedback.

## 2. Methods and subjects

### *2.1. Statistical analyses*

All statistical analyses were carried out with the Statistical Package for the Social Sciences (SPSS) version 10.0 for Windows (Release 10.0.7 [1. June 2000] Chicago: SPSS Inc.). For normally distributed data parametric methods (t-Tests, analysis of variance with repeated measurements [MANOVA], Pearson correlations, and partial correlations) were used. In case of significant deviations from the normal distribution (indicated by the Kolmogorov-Smirnov-Test), we used corresponding non-parametric methods (Mann-Whitney-U-Tests and Spearman correlations). To adjust for multiple comparisons, results were Bonferroni corrected.

### *2.2. Participants*

We studied 20 non-demented patients with idiopathic PD (11 male, 9 female) and 20 healthy control subjects (control group, CG, 14 male, 6 female). The groups were comparable with respect to gender ($Chi^2 = 0.96$, $p = 0.327$), age (PD: mean = 66.85, sd = 9.68 years; CG: mean = 64.0, sd = 7.25 years; $t = 1.05$, $p = 0.299$) and years of school education (PD: mean = 9.1, sd = 1.15; CG: mean = 9.8, sd = 1.57; $t = -1.72$, $p = 0.093$). Duration of illness in the PD group was 106.05 months (sd = 81.11). According to the criteria of Hoehn et al. [25], most of the patients were classified as stage III (median = 3, range 2–4). The mean total UPDRS score [20] was 47.25 (sd = 20.42). Disease types were: equivalence ($n = 12$), akinetic-rigid ($n = 5$), and tremor ($n = 3$). All patients were treated with typical dopaminergic medication without alteration of entrainment or dose rate for at least one month before examination. Clinically relevant depressive symptoms or signs of pathological gambling according to DSM IV criteria [3] as well as any other current or past neurological or psychiatric disease were exclusion criteria for both PD patients and healthy controls. Neither patients nor controls received financial incentives for participation. All participants were informed about the goals and procedure of the study and gave written consent to participate.

## 2.3. Methods

### 2.3.1. Neuropsychological test battery

All subjects were examined with a neuropsychological test battery including tests for the assessment of different components of executive functions, working memory, logical thinking, visuo-spatial abilities, and anterograde memory.

*General cognitive state*

General cognitive state was determined by a German version [32] of the Mini Mental State Examination (MMSE) [21] and the German version [31] of the DemTect [28]. These screening tests contain various subtests to measure decline of cognitive functions such as anterograde memory, language comprehension and production, number processing, and visuo-constructive abilities. They were also used to detect possible dementia in the PD patients as dementia was an exclusion criterion for participation in the study.

*Executive functions and working memory*

Executive functions were investigated by the Modified Card Sorting Test (MCST) [39]. In this computerised version of the task, subjects are given a total of 48 cards each showing one to four symbols of different shapes (circle, triangle, star, or square) and different colours (red, blue, green, or yellow). Subjects are asked to place the cards one by one under four different stimulus cards, presented on the top of the screen. The possible sorting rules (according to shape, colour, or number of symbols) have to be learned by the subjects using the feedback (right or wrong) of previous trials. The sorting rule changes after six consecutive correct responses. Then subjects have to shift the rule and try one of the remaining categories. The MCST assesses categorisation, set-shifting, monitoring, and a tendency to perseverate as well as cognitive flexibility and the ability to utilise feedback (see [47]).

Additionally, a controlled oral word association test (FAS-Test) [47] was administered to assess verbal fluency. Subjects have to generate as many words as possible starting with a specific letter ('F', 'A', and 'S'). Performance in verbal fluency tasks reflects speed of information processing and language abilities as well as executive functions [11]. Working memory was examined using the subtest 'digit span reverse' of the DemTect [31] in which digits (2 to 6) are orally presented and have to be repeated in the reverse order.

*Anterograde memory, logical thinking, and visuo-spatial abilities*

Verbal anterograde memory was assessed by the subtests 'word list' and 'delayed recall of the word list' of the DemTect [31]. Ten words are orally presented to the subjects for two times. Following each of both trials, subjects have to recall as many words as possible. After a delay of ten minutes, the words have to be recalled once again. For the examination of logical thinking the subtest 4 of a German intelligence test battery, the 'Leistungsprüfsystem' [49], was used. In this task participants are shown several rows of digits and/or letters and are required to detect a rule in each row. The rule must be used to eliminate one wrong item in every sequence. Subtest 7 of the 'Leistungsprüfsystem' [49] was administered to assess the ability of visuo-spatial rotation. In this subtest rows of differently rotated digits or letters are presented. Each row contains one item, which is printed mirror-inverted. This incongruous item has to be detected and signed by the participants.

### 2.3.2. The Game of Dice Task to assess decision-making

*Task description*

To examine decision-making in a gambling situation with explicit rules the Game of Dice Task [10] was used. The Game of Dice Task is based on the concepts of the Iowa Gambling Task of Bechara et al. [5] but with explicit rules for gains and losses as well as winning probabilities which are stable during the entire task duration. In the computerised Game of Dice Task, the subjects are asked to increase their imaginary starting capital (1,000 €) within 18 throws of a single virtual die. Before each throw, subjects have to guess which number will be thrown next and have to choose a single number or a combination of numbers (2, 3 or 4 numbers). Each choice is associated with specific imaginary gains and losses dependent on the probability of occurrence of choice (a single number with a winning probability of 1:6 = 1,000 € gain/loss; combination of two numbers with a winning probability of 2:6 = 500 € gain/loss; combination of three numbers with 3:6 ratio = 200 € gain/loss; combination of four numbers and a ratio of 4:6 = 100 € gain/loss). The rules and amounts of gains and losses are explicitly described in the test instruction and are visualised on the screen. Subjects are also informed that they have to make a total of 18 decisions. After each throw the gain (in case of congruence between the selected number(s) and the thrown number) or the loss (in case of incongruence between the selected number(s) and the thrown number) and the altered capital are presented visually and pointed out by two different acoustic signals for gain or loss. In addition, the number of remaining dice throws is also shown on the screen. The result of the throws are pseudorandomised (each of the six possible numbers occur three times during task performance but in a balanced order). The maximum final outcome can be 19,000 € (if the subject chooses a single number and is successful in each throw). The maximum deficit can be $-17{,}000$ € (if the subject chooses a single number and is unsuccessful in each throw).

*Analysis of game of dice task performance*

To analyse the decisions, the choices of one or two numbers (probability of winning less than 50% and high gains but also high penalties) are defined as 'disadvantageous' choices, whereas the choices of three and four numbers (probability of winning 50% and higher, low gains but also low penalties) are classified as 'advantageous' choices. For example the choice of a single number will lead to a gain of 1,000 € with a probability of 1:6 (16.67%) and will result in a loss of 1,000 € with a probability of 5:6 (83.33%). The choice of four numbers will lead to a gain of only 100 € but with a winning probability of 4:6 (66.67%) and will result in a loss of 100 € with a probability of only 2:6 (33.33%). In addition to the analysis of the frequency of disadvantageous (single number, two numbers) and advantageous (three and four numbers together) decisions, one can also analyse the frequency of choices of each alternative (single number, 2, 3, or 4 numbers).

For the analysis of feedback processing within the Game of Dice Task, it is differentiated between 'used' and 'non-used' negative feedback following a disadvantageous choice for the next decision. If subjects had chosen a disadvantageous alternative and received a negative feedback (loss of 1,000 or 500 €, dependent on the choice of a single number or two numbers) in the previous trial and then chose an advantageous alternative in the following trial, this is rated as 'used negative feedback for a decision-shift to an advantageous alternative'. In contrast, if subjects had chosen again a disadvantageous alternative following negative feedback for a disadvantageous decision the feedback was 'not used'. This procedure contains two limitations: (1) Patients and control subjects who did not make at least one disadvantageous choice during the entire task duration or those who did never receive a negative feedback following a disadvantageous decision have to be excluded from the analysis of feedback use; (2) The frequency of 'used negative feedback' has to be set in relation to the total amount of received negative feedback following a disadvantageous decision, due to the inter-individual differences in the frequency of received negative feedback.

## 3. Results

### 3.1. Results in the neuropsychological test battery

Results of the neuropsychological test battery are shown in Table 1. Even though there was a significant difference between the PD group and the CG in the total score of the DemTect, this difference is not of clinical relevance because both groups had scores clearly above the cut-off score for cognitive decline, which is 13 points. Supporting that result, all PD patients scored above the cut-off score for dementia in the MMSE (as all healthy controls also did). However, compared to the CG, PD patients scored significantly lower in most of the examined specific neuropsychological functions. Primarily, lower performance was observed in executive functions and working memory though the PD patients were on average not clinically impaired in these functions (e.g. mean t-scores above 40 in all dimensions of the MCST).

### 3.2. Main results in the game of dice task

In the Game of Dice Task the PD patients selected the disadvantageous alternatives more frequently than the control subjects (PD: mean = 10.15, sd = 4.53; CG: mean = 5.01, sd = 4.24; $t = 3.67$, $p = 0.001$) (Fig. 1). The PD patients also were in deficit at the end of the game whereas the control subjects had a positive final balance (PD: median= $-$ 3,500 €, range = $-$ 12,000 € to 2,000 €; CG: median = 700 €, range = $-$ 6,900 € to 1,800 €; U = 89.50, $p = 0.005$).

For the analysis of feedback processing in the Game of Dice Task, one patient of the PD group had to be excluded (according the mentioned procedure of feedback analysis, see Section 2.3.2) because he did not receive a negative feedback for a disadvantageous decision. Furthermore, seven control subjects could not be enrolled in the analysis because they did not make any disadvantageous decisions. Accordingly, the use of negative feedback could be analysed for 19 PD patients and 13 healthy controls. Compared to the controls, the PD patients used negative feedback for a decision-shift to an advantageous alternative less frequently (PD: mean 38.25%, sd = 26.76; CG: mean = 68.84%, sd = 31.30; $t = -2.96$, $p = 0.006$).

Furthermore, we conducted an analysis of variance with repeated measurements with 'choice' as within-subject factor and 'group' as between-subject factor to analyse differences between the PD patients and the controls regarding the frequencies of decisions for each

Table 1
Results of the neuropsychological test battery of the Parkinson's patients (PD group) and the healthy controls (control group, CG)

| Domain/test | Max | PD | | CG | | T | P |
|---|---|---|---|---|---|---|---|
| | | mean | sd[1] | mean | sd[1] | | |
| General cognitive state | | | | | | | |
| Mini Mental State Examination (MMSE) | 30 | 28.2 | (1.7) | ⩾ 27 | | | |
| DemTect (transformed total score) | 18 | 15.1 | (2.1) | 16.5 | (1.6) | −2.37 | 0.02 |
| Memory | | | | | | | |
| Subtest Word list of the DemTect immediate recall | 20 | 12.2 | (2.5) | 13.6 | (2.1) | −1.91 | 0.06 |
| delayed recall | 10 | 4.0 | (2.0) | 4.7 | (1.2) | −1.34 | 0.18 |
| Digit span reversed | 6 | 4.0 | (0.9) | 5.2 | (0.4) | −5.44 | <0.001 |
| Executive functions and information processing | | | | | | | |
| Modified Card Sorting Test (MCST) categories | t-scores[2] | 40.3 | (10.2) | 54.0 | (4.9) | −5.41 | <0.001 |
| errors | t-scores[2] | 43.0 | (8.9) | 53.2 | (1.7) | −5.03 | <0.001 |
| perseverations | t-scores[2] | 50.5 | (7.6) | 55.0 | (5.4) | −2.15 | 0.037 |
| Controlled oral word association 'F', 'A', 'S' | / | 35.0 | (15.8) | 35.7 | (14.8) | −0.14 | 0.88 |
| 'supermarket' | / | 22.8 | (5.5) | 28.2 | (4.1) | −3.52 | 0.001 |
| Subtest 4 'reasoning' of the LPS[3] | t-scores | 43.5 | (13.6) | 67.0 | (8.7) | −6.50 | <0.001 |
| Subtest 7 'visual-spatial abilities' of the LPS[3] | t-scores | 51.9 | (15.6) | na[4] | | | |

[1] sd = standard deviation.
[2] norm scores of [35].
[3] LPS = 'Leistungsprüfsystem' (German intelligence test battery, see text).
[4] na = not administered.

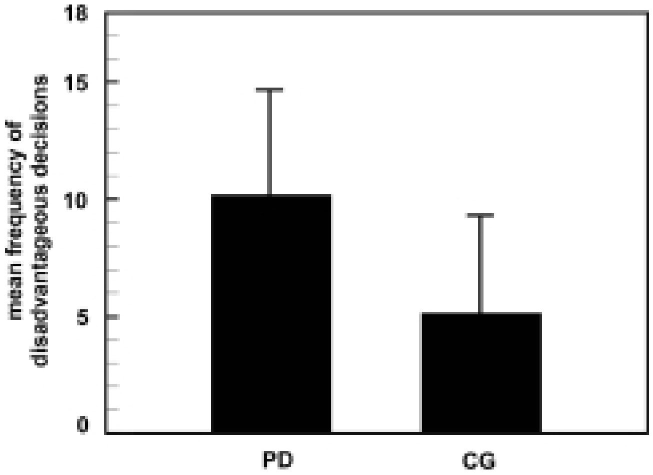

Fig. 1. Frequency of disadvantageous decisions (mean and standard deviation) in the Game of Dice Task of patients with Parkinson's disease (PD) and the healthy control group (CG).

alternative (single number, 2, 3, or 4 numbers together). There was no significant main effect for 'choice' ($F = 1.82$; $p = 0.146$). However, we found a significant interaction of 'choice' X 'group' ($F = 8.33$; $p < 0.001$). PD patients more frequently selected the most disadvantageous choice (one single number), whereas control subjects most often selected the advantageous combination of three numbers (Fig. 2). Separate between-group comparisons of selection frequency of each single alternative revealed significant differences for 'one single number' (PD: mean = 6.40, sd = 4.41; CG: mean = 1.30, sd = 1.97; $t = 4.72$, $p < 0.001$) and 'three numbers' (PD: mean = 4.05, sd = 2.68; CG: mean = 7.10, sd = 4.07; $t = -2.79$, $p = 0.008$) whereas the frequency of 'two numbers' (PD: mean = 3.75, sd = 2.48; CG: mean = 3.75, sd = 3.38; $t = 0.0$, $p = 1.0$) and 'four numbers' (PD: mean = 3.65, sd = 3.16; CG: mean = 5.85, sd = 4.64; $t = -1.74$, $p = 0.088$) did not differ between patients and controls.

### 3.3. Correlations and subgroup analyses

In both groups, the frequency of disadvantageous decisions was highly correlated with the final outcome (PD: Rho = − 0.78, $p < 0.001$; CG: Rho = − 0.59, $p = 0.007$) and with the use of negative feedback (PD:

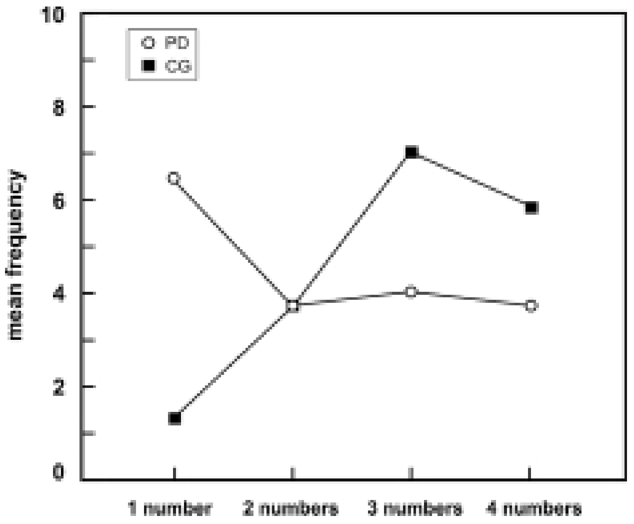

Fig. 2. Mean frequency of single alternatives in the game of dice task of Parkinson's patients (PD) and healthy controls (CG).

$r = -0.776, p < 0.001$; CG: $r = -0.650, p = 0.006$). In the PD group, the frequency of disadvantageous decisions was also correlated with performance in the Modified Card Sorting Test (MCST non-perseverative errors raw scores: $r = 0.51, p = 0.021$; non-perseverative errors t-scores according to Lineweaver et al. [35] $r = -0.460$, $p = 0.047$). All other correlations with the neuropsychological tasks failed to reach significance. Furthermore, in the PD group the correlations between Game of Dice Task performance and duration of illness ($r = -0.290$, $p = 0.215$), total UPDRS score ($r = 0.046$, $p = 0.847$), and Hoehn and Yahr stage (Rho $= -0.021$, $p = 0.931$) failed to reach significance.

To exclude the possibility that in the PD group the correlation between the use of negative feedback and the frequency of disadvantageous decisions was mediated by performance in the MCST we conducted a partial correlation analysis for the correlation between 'use of negative feedback' and 'disadvantageous decisions in the Game of Dice Task' controlled for 'MCST non-perseverative errors' (because of the strong correlation between this MCST subscore and the Game of Dice Task). This analysis still revealed a significant correlation between Game of Dice Task disadvantageous choices and use of negative feedback ($r = -0.699$; $p = 0.002$).

Beyond the analysis of correlation between performance in the Game of Dice Task and MCST, we divided the patients into two subgroups: those who were unimpaired in the MCST (non-perseverative errors, t-scores according to the normative data of Lineweaver et al. [35]) and those who were impaired. The two subgroups differed significantly regarding the frequency of disadvantageous decisions in the Game of Dice Task (impaired: $n = 9$, mean $= 12.75$, sd $= 3.32$; unimpaired: $n = 11$, mean $= 7.72$, sd $= 4.0$; $t = 2.89$, $p = 0.01$). Additionally, we divided the PD sample into subgroups according to their deficits in executive functions (t-scores of non-perseverative errors in the MCST) and disturbances in the use of negative feedback (cut-off: used negative feedback in percent below mean minus 1sd of the healthy controls included in the present study $= 37\%$). Following this procedure we defined four subgroups: (1) neither executive dysfunctions nor feedback disturbances, (2) executive dysfunctions but intact feedback processing, (3) intact executive functions but reduced feedback processing, and (4) both executive dysfunctions and reduced feedback processing. The frequency of disadvantageous decisions in the Game of Dice Task for the four subgroups of PD patients are shown in Table 2. Note that, on a descriptive level, the patients without executive deficits or reduced feedback processing performed the Game of Dice Task almost identical to healthy controls (see above). In contrast, patients who exhibited either disturbed MCST performance or reduced feedback processing were impaired in the Game of Dice Task as were patients with both deficits.

## 4. Discussion

Our primary result is that patients with PD are impaired in a decision-making task with explicit rules for

Table 2
Frequency of disadvantageous decisions in the game of dice task of the four subgroups of the Parkinson's patients

| Subgroup | n | mean (sd) |
|---|---|---|
| Neither executive deficits nor reduced feedback processing | 7 | 5.85 (3.23) |
| Executive deficits but intact feedback processing | 2 | 12.50 (0.71) |
| Reduced feedback processing but intact executive functions | 4 | 11.00 (3.16) |
| Executive dysfunctions and reduced feedback processing | 7 | 13.28 (3.77) |

gains and losses. Their deficits are strongly correlated with executive functions and emotional feedback processing, but do not correlate with any other neuropsychological function or the general cognitive status. The present findings are in accordance with previous studies on decision-making in gambling tasks with implicit winning rules (as the Iowa Gambling Task) in PD patients and other patients suffering from brain dysfunctions of the dopaminergic and/ or serotonergic system. Of particular interest is the study conducted by Thiel et al. [51] (see Introduction) which demonstrated that in PD patients Iowa Gambling Task performance was correlated with lower activation of orbitofrontal cortex compared to healthy subjects. Within the limbic loop, the orbitofrontal cortex is probably the most important region engaged in processing of negative feedback in order to avoid future negative consequences of decisions [12]. Negative feedback use is also attributed to the amygdala and to the temporopolar cortex [6], two regions which otherwise are necessary for affectional-sensory integration (cf. [36]) and which are strongly interconnected with the orbitofrontal cortex. In PD subjects these limbic circuits are affected by degeneration of nigral dopaminergic neurones and, in addition, by a decrease of serotonergic transmission from dorsal midbrain areas [30]. The importance of striatal dopamine transmission for reward processing was pointed out by a recent study of Zald et al. [54] which investigated neural correlates of reward mechanisms using ($^{11}$C)raclopride-PET in healthy subjects. Additionally, the importance of the neostriatum for habit learning was highlighted by the study of Knowlton, Mangels, and Squire [33], which found PD patients to be impaired in a probabilistic classification task. Therefore, one could assume that PD patients are impaired in implicit learning the reward-alternative and the punishment-alternative associations by using the feedback of previous decisions. This deficit might contribute to decision-making disturbances in a gambling task in these patients. Our results support the assumption that deficient functioning of the limbic loop [1, 2] in PD patients may affect decision-making in these patients in a gambling task (even in a task with explicit rules). Our patients showed lower (impaired) use of the emotional negative feedback – in terms of high losses – which was correlated with task performance. We assume that in PD patients the capacity to form stable associations between different choices and their related feedback of previous trials is affected due to the mentioned dysfunctional dopaminergic striatal-limbic circuits.

Nevertheless, beyond this interpretation of our results, we further assume that the cognitive loop and specifically the dorsolateral prefrontal cortex – as the main prefrontal region engaged in the cognitive loop – is also affected in PD patients and that this dysfunctional neural system contributes to their decision-making impairments as well. Although previous studies with different groups of frontal lobe damaged patients or subjects with frontal dysfunctions showed abnormal gambling performances (e.g. measured using the Iowa Gambling Task) in the context of intact executive functions [7,8,14,15], we did not observe such dissociation in our study. Instead, gambling performance was highly correlated with executive functions (rather indicating an association than a dissociation of decision-making and executive functions). One reason for this divergence of results might be the use of different gambling tasks, namely the Iowa Gambling Task, which offers implicit rules and the Game of Dice Task with explicit rules for gains and losses. As described by Brand et al. [10] Game of Dice Task performance probably depends on various executive functions beyond feedback processing. These comprise, for example, categorisation of the different alternatives regarding winning probability and associated amount of gain or loss, development and application of a strategy to maximise the outcome, evaluation of whether previous decisions were goal-oriented, and monitoring how many decisions need to be made by the end of the game and accordingly checking the current strategy and possibly applying another strategy. In detail, the categorisation of alternatives and their associated features are similar to the demands of the MCST. Various studies found executive dysfunctions in patients with idiopathic PD (e.g. [19,24,26,34,52]), which is in line with our data showing an impaired MCST performance in almost 50% of our PD subjects (t-score lower than 40, see above). In view of previous animal studies (cf. [29]), neuroimaging investigations on healthy subjects [40] and patient studies [22], executive functions depend primarily on dorsolateral prefrontal cortex functions (and additionally on other cortical regions such as posterior parietal lobe as well as parts of basal ganglia). In accordance with the high correlation between Game of Dice Task and MCST performance (as mentioned above), executive deficits resulting from disturbances of dorsolateral prefrontal cortex functions in PD [34] can probably also lead to the poor decision-making abilities measured by a gambling task with explicit rules. Further evidence for this interpretation can be derived from the subgroup analysis, which demonstrated, on a descriptive level, a tendency for unimpaired Game of Dice Task performance in patients with neither executive deficits nor reduced feedback processing. In contrast, patients who were deficient in one of these components or in both showed similar high frequencies of disadvantageous decisions.

Based on the reported correlations we conclude that in PD patients, Game of Dice Task performance might be impaired due to deficits in two domains: executive functions and emotional feedback processing. Correspondingly, decision-making deficits of patients with PD may be based on disturbances of fronto-striatal loops connecting the basal ganglia with both limbic and orbitofrontal projection cortices as well as with the dorsolateral prefrontal cortex. Further research might investigate the neural correlates of decision-making impairments of PD patients in an explicit gambling situation using functional brain imaging techniques. Additionally, future studies might examine PD patients with both the Iowa Gambling Task and the Game of Dice Task to compare their performances in both implicit and explicit gambling tasks.
